# Heveenoid and Rubber Dam

**Published:** 1881-06

**Authors:** 


*Heveenoid and Rubber Dam.*—A rather highly colored article on the merits of this new compound of rubber, sulphur and camphor was published a few months ago. The manufacturers sent the writer a piece of the elastic goods made for the uses to which rubber dam is devoted. With it came a letter stating that "with regard to the superior durability of soft Heveenoid over soft rubber, we would say that the sulphur in Heveenoid enters into combination with the camphor, forming a sulphide of camphor, which will remain incorporated in and with the rubber in all times to come, and will keep the fabric, in any length of time, as perfect and elastic as when it was made." With this official guarantee this piece of dam, which was certainly very elastic and tough, was laid aside. In one month it was the most brittle and rotten piece of elastic rubber we ever handled, utterly worthless and flying to pieces like a rotten rag. It may be that this was an exceptionally poor piece, and that the quality is now improved, but this particular specimen was particularly worthless.

While on this subject, the best method of keeping rubber dam is of interest. It is an article which soon deteriorates as usually kept, and it is often the case that the beginning of a yard of the most excellent dam turns out toward the end very indifferent indeed. For some time we kept it cut up into suitable pieces, between the leaves of an old atlas, thereby keeping the air from it, and preserving it somewhat; but this was only retarding the decay. Inquiry of parties engaged in selling it developed the fact that the manufacturers of rubber dam kept it in large quantities in good condition, by storing it in *damp cellars.* As it was inconvenient to run down into the cellar for a piece of dam, a damp receptacle was improvised by taking a stone jar, and making a diaphragm of wood across it, half way from the bottom. This diaphragm was pierced with auger holes, and an inch of water poured in, which by its evaporation made a moist atmosphere in the jar. The rubber was laid on the top of the diaphragm, above the water, and the stone cover being put on, the results are quite satisfactory. The rubber seems to keep much longer in this way than by any method heretofore adopted.

It may be of value to some to know that small patches may be made to cover holes in rubber dam, by sticking them on with gutta percha cement. Benzine is as good a solvent of gutta percha for this purpose as anything else. H.

